# Acute Kidney Injury in a Previously Healthy 56-Year-Old Male Following a Direct Visual Internal Urethrotomy of a Bulbar Stricture

**DOI:** 10.7759/cureus.59310

**Published:** 2024-04-29

**Authors:** Charles D Calenda, Cameron R Toohey, Madeline Levy, Nisha Vanmali, Jaspreet Ubhi, Noshi Ishak, Stephen D Marshall

**Affiliations:** 1 College of Osteopathic Medicine, University of New England, Biddeford, USA; 2 Department of Internal Medicine, Concord Hospital - Laconia, Laconia, USA; 3 Department of Nephrology, Concord Hospital - Laconia, Laconia, USA; 4 Department of Urology, Concord Hospital - Laconia, Laconia, USA

**Keywords:** reflex anuria, bulbar stricture, direct visual internal urethrotomy, anuria, acute kidney injury

## Abstract

Acute kidney injury (AKI) is a frequent finding in acutely ill and hospitalized patients arising from various etiologies. Anuric AKI, a more pronounced form of AKI in which less than 100 cc of urine is produced per day, is most frequently encountered in hospitalized, septic, and post-surgical patients, often secondary to shock or bilateral urinary tract obstruction. The development of anuric AKI in previously healthy patients after outpatient urological procedures presents a unique challenge to physicians, as many outpatient procedures require the routine perioperative administration of multiple nephrotoxic medications. Further complicating this clinical scenario, some surgical procedures that intrinsically involve iatrogenic injury to the kidney, ureter, bladder, or nearby organ can rarely lead to a phenomenon known as reflex anuria, an anuric state typically associated with AKI. Here, we report an unusual case of a previously healthy 56-year-old male who developed anuric AKI two days after direct visual internal urethrotomy (DVIU) for the treatment of a bulbar stricture. Non-contrast CT revealed no signs of an obstructive process, and laboratory findings supported an intrarenal cause of AKI. Consideration was given to non-steroidal anti-inflammatory drugs (NSAID)-induced nephrotoxicity, gentamicin-associated acute tubular necrosis, and propofol infusion syndrome, in addition to their potential synergistic effects. We also explore this as the first reported case of reflex anuria occurring at the level of the bulbar urethra, as most cases have involved direct injury to the kidney or ureter. Over the course of 10 days, our patient responded well to treatment with supportive measures and dialysis, with his vomiting, electrolyte abnormalities, renal state, and anuria eventually improving.

## Introduction

Urethral stricture, most often formed in the bulbar segment, involves urethral narrowing due to the presence of scar tissue, resulting in dysfunctional voiding and potentially severe implications for the overall health of the urinary tract [1,2]. Bulbar urethral stricture etiologies can be divided into traumatic, most often due to luminal insult, and non-traumatic, which commonly includes congenital, infection-related, and iatrogenic manipulation [3]. Existing urethral strictures result in complications in around 90% of men, with the leading primary signs and symptoms including reduced urine flow, urinary urgency, dysuria, incontinence, hematuria, and suprapubic pain [4]. When contemplating treatment options, the severity of symptoms, length and location of stricture, and patient preferences must be considered [4]. 

Retrograde urethrography can effectively provide visualization of the urethral stricture, and cystoscopy can also be utilized to effectively diagnose urethral stricture while allowing for simultaneous therapeutic dilation [5]. Surgical treatment, particularly direct visual internal urethrotomy (DVIU), of urethral stricture can provide therapeutic relief of symptoms [5]. For cold knife urethrotomy, a meta-analysis found that success rates ranged from 19.6% at the four-month mark to as high as 98.2% at the 31.5-month mark, with longer intervals between surgery and re-evaluation showing a favorable trend toward marked improvement [6].

The high success rates seen in DVIU are in stark contrast to the low rate of complications experienced after the procedure, previously reported to be around 6.5% [6]. After mechanical fixation of their stricture, patients may experience hematuria, incontinence, infection, impotence, and stricture recurrence [4]. While physicians and their patients know of such predictable complications, patients may experience unanticipated complications. Here, we report the case of anuric acute renal failure in a 56-year-old previously healthy male two days after DVIU with dilation for a 2 cm bulbar stricture. To the best of our knowledge, there are no cases of anuric acute kidney injury (AKI) linked to DVIU. In this current report, we discuss the most likely etiologies of anuric AKI in our patient, which include ketorolac, propofol, and gentamicin, while entertaining a diagnosis of exclusion as a possible cause of our patient's anuric AKI.

## Case presentation

Our patient is a 56-year-old male who presented for a workup of microscopic hematuria with concurrent complaints of recurrent urinary frequency and urgency over the past few years, in addition to a recent urinary tract infection. Past medical history included hypertrophic obstructive cardiomyopathy, while surgical history was notable for a urethral biopsy at 17 years old. Family and psychosocial history were unremarkable. Previous renal function labs were within normal limits, including GFR (glomerular filtration rate), blood urea nitrogen (BUN), and creatinine. Prostate-specific antigen levels were normal at 0.99 ng/mL.

The most recent urinalysis approximately one month prior revealed three to five red blood cells/high-powered field, consistent with microscopic hematuria. Initially, a cystoscopy was performed to rule out insidious causes of microscopic hematuria, which revealed a bulbar urethral stricture, and advancement was aborted to avoid urethral damage. Follow-up was scheduled for cystoscopy and retrograde urethrogram to characterize the bulbar stricture further and assess the opportunity for surgical options, including DVIU, urethral dilation, and formal urethroplasty. In the context of recurring symptoms, the patient decided to pursue DVIU with steroid injection. On the day of the procedure, the patient was anesthetized with propofol (200 mg/20mL) and perioperatively received ketorolac (30 mg/mL), ampicillin (2 g), and gentamicin (150 mg), in addition to other standard medications (Table 1).

**Table 1:** DVIU perioperative medications Perioperative DVIU medications with their respective doses, routes, and frequencies. DVIU: direct visual internal urethrotomy

Medication	Dose	Route	Frequency
Lidocaine	2%	Topical	Once
Gentamicin	150 mg	IV	Once
Triamcinolone (Kenalog-40)	400 mg	Intralesional	Once
Iopamidol (Isovue-300)	100 mL	IV contrast	Once
Dexamethasone	4 mg/mL	Injection	Once
Ondansetron	4 mg/2mL	IV	Once
Propofol	200 mg/20mL	IV	Once
Fentanyl	100 mcg/2mL	IV	Once
Ketorolac	30 mg/mL	IV	Once

**Figure 1:**
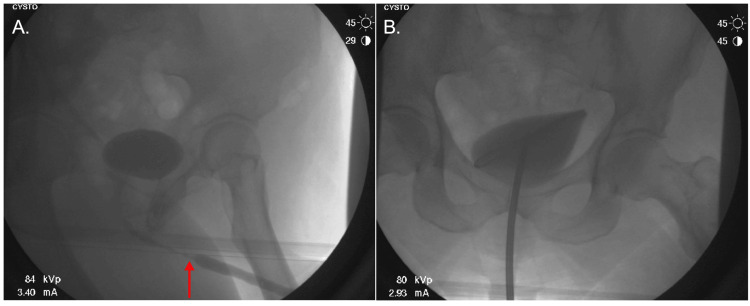
Retrograde urethrogram before and after DVIU with the placement of the catheter (A) Retrograde urethrogram revealing decreased contrast signal proximal to the urethral stricture (red arrow). (B) Anteroposterior view confirming successful resolution of the stricture after DVIU with proper placement of the catheter. DVIU: direct visual internal urethrotomy

The stricture (Figure 1A) was serially dilated with Amplatz dilators. Kenalog-40 (400mg/10mL) was injected into three different regions of the stricture. After a rigid French cystoscope was used to direct a 0.035-inch sensor wire through the stricture, confirmed through cystogram and fluoroscopy (Figure 1B), Amplatz dilators from 14 French to 22 French were utilized. A 21 French rigid urethrotome was used for a DVIU at the 12:00, 5:00, and 7:00 locations, with subsequent injection of Kenalog-40 (400 mg/10mL) using NephroMax (Boston Scientific, Marlborough, Massachusetts, United States) at these sites. Isovue (100 cc) was utilized to visualize the bladder and the sensor wire. Blood secondary to the urethrotomy limited visualization during cystoscopy. There were no obvious bladder tumors visualized during the procedure, and both right and left ureteral orifices were in the normal orthotopic position. Following the procedure, the patient was awoken without any complications, and the plan was to remove the Foley catheter in one week at follow-up.

**Figure 2:**
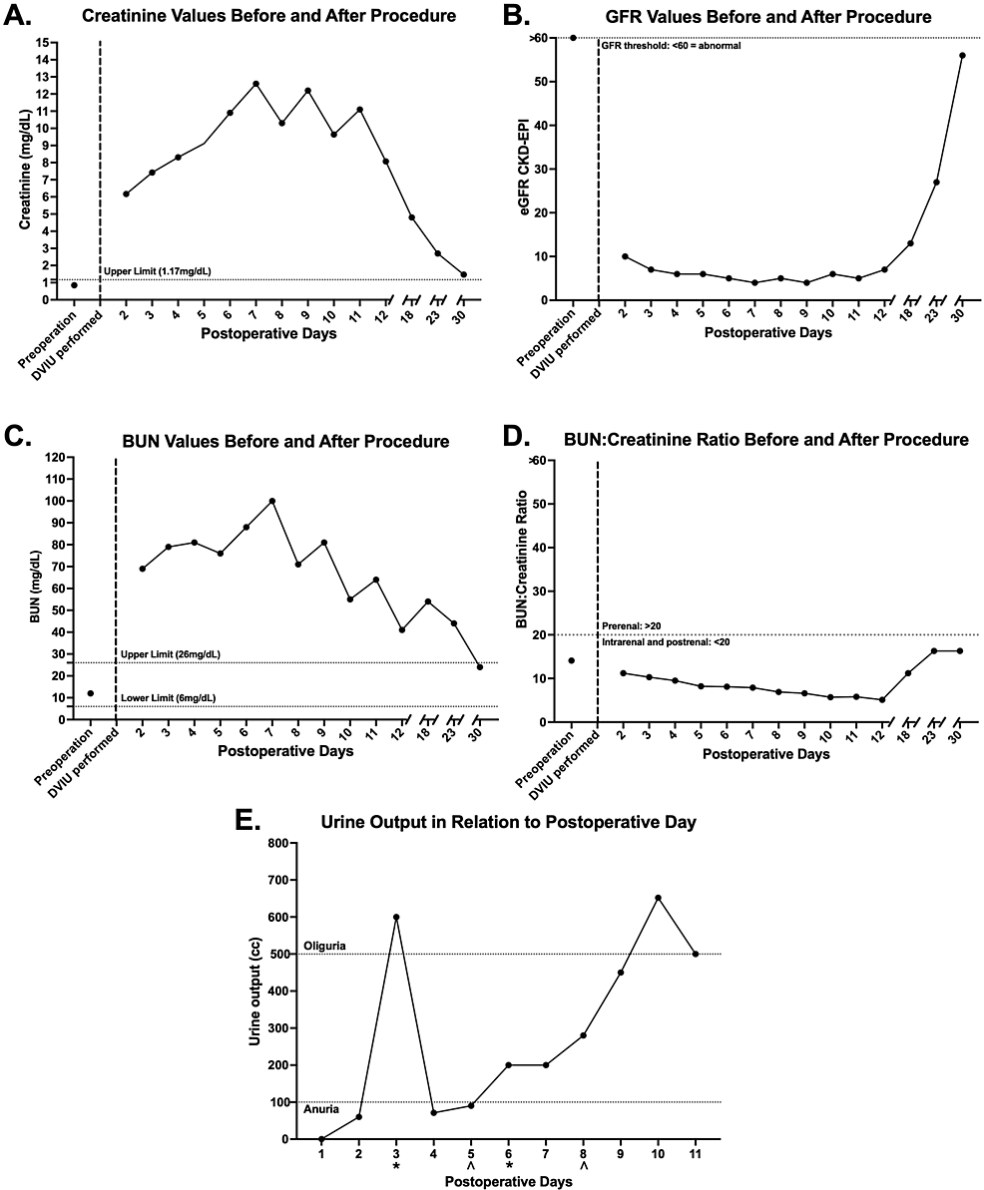
Creatinine (A), GFR (B), BUN (C), BUN: creatinine ratio (D), and UOP (E) in relation to days after DVIU Preoperative baseline values were measured approximately three months before the development of anuric AKI. Creatinine levels (A) and BUN levels (C) remained elevated throughout the hospital course and markedly improved after discharge, while GFR levels (B) improved after discharge. The BUN: creatinine ratio remained below 20 throughout the admission (D). Furosemide stress test (*) and dialysis (^) were administered on multiple days, and UOP increased after the initial stress test (E). GFR: glomerular filtration rate; BUN: blood urea nitrogen; UOP: urine output; DVIU: direct visual internal urethrotomy

On postoperative day (POD)-1, the patient began experiencing intractable vomiting and episodes of retching. On POD-2, the patient presented to the emergency department (ED) complaining of nausea, vomiting, retching, decreased oral intake and reported no urine output (UOP) since POD-1. Vital signs and physical examination were both within normal limits. The patient presented with a WBC count of 15.01 x 10^3^/mcl and a temperature of 36.5°C. Serum levels of creatinine (Figure 2A) and BUN (Figure 2C) were markedly elevated, while GFR (Figure 2B) was significantly decreased, consistent with a diagnosis of AKI. UOP on POD-1 was 0 cc/day per the patient's history (Figure 2E). UOP was measured by the Foley catheter thereafter, and measurements from POD-2 through POD-5 were 60 cc/day, 600 cc/day, 71 cc/day, and 90 cc/day (Figure 2E), consistent with anuria. 

**Figure 3:**
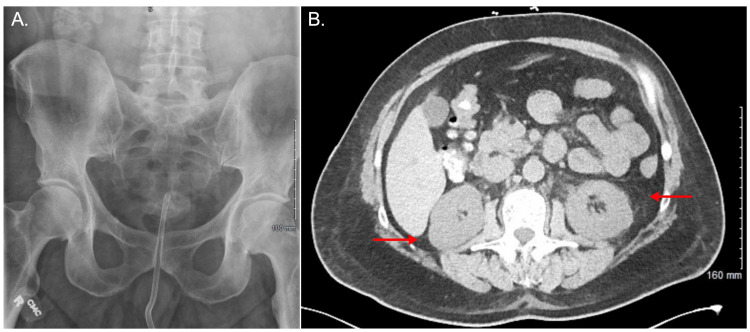
KUB and abdominal CT on presentation to the emergency department (A) KUB confirming catheter placement with no significant findings. (B) CT showing mild perinephric fat stranding bilaterally (red arrows) without signs of urinary obstruction. KUB: kidney, ureter, and bladder radiography

Kidney, ureter, and bladder (KUB) radiography and renal ultrasonography were unremarkable (Figure 3A), while non-contrast CT (Figure 3B) revealed mild perinephric fat stranding without signs of obstruction. Early in the course of our patient's AKI, a furosemide stress test was employed, and the patient's UOP increased (Figure 2E). Creatinine levels were markedly elevated and continued to trend higher, along with BUN levels, prompting the initiation of dialysis on POD-5 and POD-8 (Figure 2E). Further workup during the patient's hospital stay included creatine kinase (CK) measurements, which were within the normal range on POD-4 and POD-6.

Laboratory values were trended over time, revealing stabilization of the patient's electrolyte values, anion gap, albumin levels, protein levels (Table 2), and renal function parameters (Figure 2). Urinalysis findings were also assessed throughout the patient's stay (Table 3). On POD-12 and the 10th day of hospitalization, the Foley catheter was removed. The patient was subsequently discharged, as electrolytes were adequately controlled; he produced 500 cc of urine and was clinically stable. Following discharge on POD-12, he presented to the ED on POD-13 complaining of hematuria with noticeable blood clots, which was triaged and found to be manageable in the outpatient setting. By POD-30, further monitoring in the outpatient setting revealed that the patient's GFR returned to near-normal values while BUN was within normal limits (Figure 2). 

**Table 2:** Electrolyte, anion gap, glucose, albumin, and protein serum values in relation to POD Na^+^, Cl^-^, anion gap, K^+^, Ca^2+^, glucose, albumin, and protein serum results from baseline to POD-30. Baseline laboratory values were obtained for routine outpatient care approximately three months prior to the presentation. The patient was discharged on POD-11, and values obtained thereafter were measured in the outpatient dialysis setting. Expected values and parameters are in parentheses under each respective laboratory finding. Na^+^: sodium ion; Cl^-^:chloride ion; K^+^ :potassium cation; Ca^2+^: calcium cation; POD: postoperative day

POD	Na^+ ^(mmol/L)	Cl^- ^(mmol/L)	CO_2 _(mmol/L)	Anion Gap	K^+ ^(mmol/L)	Ca^2+ ^(mg/dL)	Glucose (mg/dL)	Albumin (g/dL)	Protein (g/dL)
-	Normal: (136-143)	Normal: (101-111)	Normal: (21-32)	Normal: (2-11)	Normal: (3.5-5.1)	Normal: (8.3-0.1)	Normal: (70-99)	Normal: (3.1-4.9)	Normal: (6.5-8.4)
Baseline	140	110	24	10	4.0	8.7	118	3.2	6.9
2	133	99	20	14	4.7	9.3	114	3.4	6.7
3	132	101	14	12	4.7	8	107	2.8	5.9
4	133	98	19	12	4.1	8.3	114	2.9	6.3
5	129	96	21	11	4.3	7.8	117	-	-
6	130	94	21	15	4.6	8.2	108	-	-
7	127	90	20	17	4.9	8.3	95	-	-
8	129	93	21	15	4.7	8.2	104	-	-
9	127	91	20	16	4.4	8	102	-	-
10	132	93	24	15	4.5	8.6	101	-	-
11	131	92	26	13	4.1	8.5	103	-	-
12	133	96	27	10	4.0	8.1	93	-	-
14	136	100	25	10	3.9	8.6	99	3.4	7.3
18	138	106	27	8	3.5	8.8	116	3.6	7.7
23	138	101	27	13	3.2	8.8	100	4.1	7.2
30	143	108	26	12	3.5	9.6	99	3.6	7.8

**Table 3:** Urine dipstick and microscopy results from baseline, at presentation, and upon second presentation for hematuria Urine findings including color, clarity, pH, WBC count, bacteria, nitrites, red blood cells, occult blood, renal epithelial cells, and protein were obtained at baseline (one month prior), at presentation for anuric AKI (POD-2), once during the hospital stay (POD-9), and upon second presentation to the emergency department for hematuria and blood clots in the urine (POD-13). Expected values and parameters are in parentheses under each respective laboratory finding. POD: postoperative day; AKI: acute kidney injury

POD	Color	Clarity	Specific gravity	pH	WBCs	Bacteria	Nitrites	RBCs	Occult blood	Renal epithelial cells	Protein
-	Normal: (Yellow)	Normal: (Clear)	Normal: (1.005-1.030)	Normal: (4.6-8.0)	Normal: (0-2/HPF)	Normal: (None seen)	Normal: (Negative)	Normal: (0-2)	Normal: (Negative)	Normal: (None seen)	Normal: (Negative (mg/dL))
Baseline	Not measured	Not measured	Not measured	Not measured	0-2 /HPF	+	Not measured	0-2 /HPF	Negative	None seen	Negative
3	Dark yellow	Slightly cloudy	1.015	6.5	3-5/HPF	++	Negative	21-50	Large	None seen	>=300
9	Dark yellow	Clear	1.01	6.5	6-10/HPF	None seen	Negative	6 to 10	Large	Present	>=300
13	Red	Cloudy	1.01	8	Present	Present	Positive	>100/HPF	Large	None seen	100

## Discussion

Bulbar strictures can cause significant genitourinary symptoms in patients that often warrant treatment, and the effectiveness of DVIU with the low risk of complications makes the procedure a feasible option [6]. The current case study highlights a unique presentation of anuric renal failure after DVIU. Here, we explore common prerenal, intrarenal, and postrenal etiologies of AKI, focusing on imaging modality results and medications known to cause AKI, specifically ketorolac, gentamicin, and propofol. A diagnosis of exclusion, namely reflex anuria, is entertained as a possible cause of the patient's presentation.

Common prerenal etiologies of AKI were explored, including hypovolemia, hypotension, and drug-induced renal vasoconstriction. Although our patient was vomiting, he remained hemodynamically stable with a BUN to creatinine ratio below the widely acknowledged threshold of 20:1 typically found in prerenal azotemia [7]. Postrenal obstructive causes of AKI were also investigated, such as calculi, renal vein thrombosis, and neurogenic bladder. Though the patient was anuric, KUB and CT imaging failed to reveal any blatant obstruction, hydronephrosis, extravasation of contrast, or other evidence of bladder or urethral injury. The only significant finding on CT imaging was mild perinephric fat stranding, suggestive of renal inflammation. 

Presentation of AKI postoperatively remains a common complication due to intraoperative anesthetic use, which was a concern considering the patient's perioperative propofol infusion. Though less likely to cause AKI than other anesthetics, propofol infusion syndrome can result in a triad of rhabdomyolysis with renal failure, metabolic acidosis, and unexplained myocardial failure [8]. Kidney injury due to propofol typically develops during instances of prolonged treatment, primarily reported in ICU patients concurrently receiving catecholamines or steroids [8]. These patients usually undergo propofol infusion for more than 48 hours and have a high anion gap metabolic acidosis and markedly elevated CK levels [9]. This is significantly different from our patient's one-off exposure to propofol intraoperatively, mild anion gap metabolic acidosis of 14 mmol/L, and CK levels measured on two separate days that were within normal limits (47, 108 U/L).

Ketorolac, a non-steroidal anti-inflammatory drug (NSAID) that our patient received perioperatively, is a common cause of renal injury mediated by two mechanisms, namely hemodynamic instability and acute interstitial nephritis (AIN) [10]. NSAID-induced AKI secondary to hemodynamic instability usually occurs in patients with predisposing factors, and symptoms should not persist for more than one to three days after discontinuation of the NSAID [10]. AKI persisting or progressing after discontinuation of NSAIDs implies a different etiology [10]. Our patient tolerated ketorolac previously on multiple occasions, generally lacked the characteristics putting him at risk for NSAID-mediated AKI, and had creatinine levels that did not decrease by 0.3 mg/dL within the first 72 hours, meeting the criteria for nonresolving AKI [11]. While we cannot rule out that ketorolac precipitated the development of our patient's AKI, it is doubtful considering that our patient received a one-off dose, previously tolerated exposure to ketorolac, and had nonresolving AKI. The second form of AKI induced by NSAIDs, AIN, can present with rash, eosinophilia, fever, arthralgias, back pain, and polyuria [12]. Considering that our patient had anuric AKI, lacked all of the signs and symptoms of AIN, and was without urinalysis (UA) findings notable in AIN, AIN is an unlikely cause of this patient's presentation.

An additional medication that our patient received perioperatively was gentamicin (150 mg), an aminoglycoside associated with acute tubular necrosis that occurs due to an accumulative effect eight to 10 days after administration [13], in stark contrast to the onset of one to two days to AKI in our patient. In a systematic review assessing the adverse effects of a single dose gentamicin by Hayward et al., the authors found that all studies that reported a statistically significant increase in AKI from a single dose of gentamicin were in orthopedic surgery patients [14]. Another study by Cobussen et al. found that patients with sepsis presenting to the ED who received a single dose of gentamicin (5 mg/kg), a similar dose to the one received by our patient, did not have a higher risk of developing AKI [15]. While the studies mentioned are conflicting, likely due to dissimilarities of the patients' presenting conditions and demographics, it is notable that a single dose of gentamicin has been shown to be tolerated amongst some of the most critically ill patients [15]. Seeing that our patient was a previously healthy male without predisposing factors who developed anuric AKI one to two days after DVIU, gentamicin alone is unlikely to have caused our patient's condition. 

Sufficient evidence exists to support that propofol, ketorolac, and gentamicin were unlikely independent causative factors precipitating the development of anuric AKI in our patient. A more plausible theory is that the medications above could have collectively led to the development of anuric AKI in our patient. A literature search on the combined effects of propofol, ketorolac, and gentamicin revealed no reports in humans. However, studies on the combined effects of gentamicin and ketorolac exist in other animals. Experiments on rats showed a synergistic effect in renal toxicity in gentamicin and ketorolac after seven doses over four days of treatment, with measurements taken on baseline day zero and on day three [16]. It is worth noting that comparisons of the current case to the studies mentioned above are limited, as the studies were done on rats, and our patient received a one-off combined treatment of gentamicin and ketorolac. 

The idea that nephrotoxic drugs independently are the driver of our patient's AKI is largely incompatible with the prevailing clinical literature, given the patient's presenting anuric state and previous state of good health. In a study of 203 patients with AKI stratified by anuric, oliguric, and non-oliguric states, only one out of 13 patients treated with nephrotoxic drugs or toxins developed anuric AKI [17]. Most importantly, from a clinical perspective, anuria is generally narrowed down to two causes: shock and bilateral urinary tract obstruction [18]. Less common conditions that involve the kidneys, such as anti-glomerular basement membrane antibody disease, bilateral renal artery stenosis, glomerulonephritis, hemolytic-uremic syndrome, and kidney cortical necrosis, can cause anuric AKI [18]. Knowing the unique presenting signs and symptoms, lab findings, and imaging features that these conditions present with, they are unlikely causes of our patient's anuric AKI.

In the context of the patient's recent DVIU and his anuric state, we explored the differential diagnosis that can lead to complete anuria following injury to the genitourinary system. This led us to an equally interesting and rare condition known as reflex anuria, initially described by Hull et al. as the cessation of urine output from both kidneys due to irritation or trauma to one kidney or its ureter, or severely painful stimuli to other organs [19]. The majority of cases reported on reflex anuria thus far have been from ureteral injury or aggravation, generally from ureteral catheterization or bilateral pyelogram [20-22]. 

Multiple mechanisms may explain how injury to the ureter could lead to anuric AKI, including a ureterorenal reflex, in which irritation or manipulation of the ureters generates a reflex of renal arteriolar vasoconstriction through sensory fibers [23]. Underlying neurological signaling pathways may be why reflex anuria is possible after injury to the urethra. While the histological profile of the bulbar urethra differs from the upper genitourinary urothelium, crosstalk may occur between these structures through shared neural pathways. Considering that the superior hypogastric plexus has sympathetic fibers connecting to both the pelvic and aorticorenal plexuses, which innervate the urethra [24] and kidney [25], respectively, this common neural pathway may explain how an injury to the urethra could trigger a reflex sympathetic response to the ureters and kidneys.

At its core, the current AKI case meets the criteria for the definition of reflex anuria: our patient had "cessation of urine output from both kidneys" after "irritation or trauma" to "other organs" [19]. It is worth pointing out that we were able to monitor our patient's UOP consistently from POD-2 with a Foley catheter. Our patient was anuric on POD-1-POD-5, except POD-3 after a trial of loop diuretics. The length of anuria in our patient agrees with findings from Kanno et al. showing that anuria in reflex anuria typically resolved over three to seven days [20]. Over the hospital course, the patient was able to produce urine again and was subsequently discharged.

## Conclusions

In light of our previously healthy 56-year-old male patient's uncommon presentation of an anuric state, unique causes of AKI were assessed beyond the scope of typically encountered clinical scenarios. After weighing all of the evidence, the literature and evidence support that our patient's presentation either resulted from a combination of medications received or due to reflex anuria secondary to urethral insult from DVIU. To the best of our knowledge, after a complete literature search, this would either be the first report to describe a presentation of anuric AKI in a previously healthy male following a one-off combination of the received medications or the first case to describe reflex anuria in which an insult occurred at the level of the bulbar urethra.
